# Do Magnetic Fields Have a Place in Treating Vascular Complications in Diabetes?

**DOI:** 10.7759/cureus.24883

**Published:** 2022-05-10

**Authors:** Harvey N Mayrovitz, Raneem Maqsood, Aneil S Tawakalzada

**Affiliations:** 1 Medical Education, Nova Southeastern University Dr. Kiran C. Patel College of Allopathic Medicine, Davie, USA; 2 Medical School, Nova Southeastern University Dr. Kiran C. Patel College of Osteopathic Medicine, Fort Lauderdale, USA; 3 College of Osteopathic Medicine, Nova Southeastern University Dr. Kiran C. Patel College of Osteopathic Medicine, Fort Lauderdale, USA

**Keywords:** skin blood flow, diabetic foot ulcers management, smf and pmf magnetic intervention, pemf, painful neuropathy, ­wound healing, diabetes, magnetic therapy

## Abstract

The use of electromagnetic field therapy (EMFT) is a non-invasive, potential alternative or complementary choice in the treatment of wounds, chronic pain, neuropathy, and other medical conditions, including tissue repair and cell proliferation. Static magnetic fields (SMFs) have been reported to increase microcirculatory blood flow by mediating vasodilation via nitric oxide. Studies report that SMF exposure causes homeostatic, normalizing effects on the vascular tone that may have beneficial effects in situations where tissue perfusion is limited, such as may be present in diabetes. Pulsed electromagnetic fields (PEMFs) have also shown promise in treating diabetic wounds by improving wound healing rates and other attributes. Our purpose was to critically review prior applications of EMFT for relevancy and effectiveness in treating diabetic complications. The goal was to provide information to allow for informed decisions on the possible use of these modalities in the treatment of persons with diabetic complications. The focus was on the following major areas: wound healing, neuropathy, blood glucose control, blood flow, inflammation and oxidative stress.

## Introduction and background

Over the past 40 years, the number of diabetes mellitus diagnoses has nearly quadrupled from 108 million in 1980 to 422 million in 2014 [1]. Complications of diabetes include neuropathy, foot wounds, delayed or nonhealing wounds, microvascular deficits, and other organ system issues [2-4]. Diabetic foot ulcers can be a serious complication of the condition and, if left untreated, can lead to severe infection, gangrene, and, in some instances, death [5]. More than one million diabetic patients undergo lower limb amputation per year, which totals to nearly 50-70% of all limb amputations performed [6]. As global cases of diabetes continue to rise, their complications follow this trend and continue to worsen morbidity and mortality. Diabetic wounds are sometimes challenging to treat and control, and as such, there is an important need for adjunctive and efficient management protocols. Electromagnetic field therapy (EMFT) is a non-invasive possible alternative or complementary choice to treat wounds, chronic pain, diabetic neuropathy, and other medical conditions, including tissue repair and cell proliferation [7-10]. Static magnetic fields (SMFs), derived either from magnets or from electrical devices carrying non-time-varying currents, are one form of EMFT that has been reported to have a variety of effects, including an increase in microcirculatory blood flow by mediating vasodilation via nitric oxide (NO). Diabetic neuropathy, a major risk factor for diabetic foot ulceration, is associated with impaired blood flow, causing inadequate tissue perfusion and potentially causing ischemia [11]. Studies have reported that SMF exposure may also have homeostatic, normalizing effects on the vascular tone that may have beneficial effects in situations where tissue perfusion is limited, such as in diabetic neuropathy [11].

While wound debridement, antibiotics, revascularization, and off-loading of plantar ulcers are the current standard of care treatments, costs and other factors sometimes cause delayed treatment, and it has been suggested that SMF might be a non-invasive, affordable diabetic wound treatment tool that works by suppressing inflammatory cytokines and accelerating wound closure and revascularization [6]. Pulsed electromagnetic fields (PEMFs) have also been suggested to be a possible treatment for diabetic wounds [12,13]. Thus, there is sufficient preliminary information to further investigate the potential role of such EMFT types with respect to diabetes complications.

## Review

Our purpose was to critically review and evaluate prior applications of EMFT with respect to relevancy and effectiveness in treating complications of diabetes with the overall goal of providing information that will allow for informed decisions as to the use of these modalities.

Search strategy

The following databases were searched for peer-reviewed published articles in the English language: PubMed, Biomed Reference Collection: Comprehensive, CINAHL Complete, Embase, and Web of Science. The main search was for articles with the word magnet or magnetic in the title and diabet* anywhere in the article text. The asterix (*) served as a wildcard. To provide a focused search, any paper that contained the following terms within its text was excluded from the search: “resonance,” “transcranial,” nano*, particle*, capsule*, magnetize*, and magnetite. This yielded 102 articles satisfying the search criteria. The titles and abstracts were reviewed for relevance to EMFT-related healing, which reduced this number to 39 relevant articles that were reviewed in depth. The bibliographies of these articles were reviewed, and an additional 14 articles were identified. Finally, articles relevant to the potential role of EMFT-related impacts on blood flow were included, yielding a total of 71 articles in the present review.

Presentation of results

Effects of EMFT, either SMF or PEMF, are presented for each of the following five major impact categories: wound healing, diabetic neuropathy, blood glucose, blood flow, and inflammation and oxidative stress.

Wound Healing

Streptozotocin (STZ) injections are often used to induce diabetes in mice and rats for research purposes [14]. Using this model, the effect of a SMF on diabetic wound healing in rats was evaluated [15]. In this study, rats were assigned to one of three groups: diabetic wound + sham treatment (n=16), diabetic wound + SMF treatment (n=16), and 16 non-diabetic control rats. A 1.5 cm diameter circular wound was created on the dorsum of all rats. Wounds were then treated with a wound dressing alone (control), a wound dressing plus a magnet (180 mT), or a wound dressing plus a sham magnet. Wound areas were measured on days 5, 12, and 19 after wounding to measure the healing rate and healing time. At each measurement, control non-diabetic animals had a statistically significant (p<0.05) greater healing rate vs. either diabetic group. However, the SMF treated group had a greater healing rate vs. the sham-treated group (p<0.05). The time for complete wound closure was less in the SMF-treated group (20 days) vs. the sham-treated group (27 days, p<0.05).

A similar study investigated the impact of a 230 mT SMF magnet vs. sham-magnet treatment on wound healing rate and time-to-heal in 20 STZ-induced diabetic rats [10]. Ten rats wore magnets over the wound and 10 wore sham magnets continuously for 21 days after the creation of a 2 cm diameter back wound. An additional non-diabetic control group that wore neither the magnet nor sham was included. The wound area was measured on days 7, 14, and 21; however, rats were continuously followed until complete wound closure. It was reported that at each measurement day, control non-diabetic rats healed faster than either diabetic group but that the SMF-treated healing rate was faster than for sham-treated animals (p<0.05). Full wound closure occurred after 22.3 ± 2.5 days in the non-diabetic controls, 29.5 ± 3.8 days in SMF-treated, and 36.5 ± 4.4 days in sham-treated, showing significant differences (p<0.05). SMF treatment effects were also evaluated in the STZ-induced diabetic mouse model after causing 8-mm punch biopsy wounds on the animal’s back [6]. The mice were placed in cages with magnetic or non-magnetic plates. The magnetic plates had 24 embedded magnets, each with a surface field of 0.6T, with adjacent magnets having alternate poles facing the plate surface. Percentage wound area closure was assessed on days 3, 7, and 14 post-wounding in eight SMF-treated and eight non-treated diabetic mice. Wound closure, measured at each of the measurement days, was reported to be significantly greater for the SMF-treated mice (p<0.05). Other measurements made in parallel suggest that the improved healing rate may be related to the upregulation of inflammatory gene expression modifying macrophage function during the healing process.

In addition, PEMF effects on wound healing have also been evaluated in STZ-induced diabetic and non-diabetic mice [16]. Skin flaps were created on the backs of 24 mice; 12 were STZ-induced diabetic mice and 12 were non-diabetic controls. Six of each group were treated with PEMF that consisted of 4.5 ms pulses of 1.2 mT peak at 15 Hz using a commercial bone-healing device (EBI, Parsippany, New Jersey, USA). The other half of each group was not treated with PEMF. The treated mice were exposed to PEMF while in their cages for eight hours a day for 14 days. Wounds were examined and measured until full wound closure. PEMF-treated mice, whether diabetic or control wild-type mice, healed at a faster rate and achieved full wound closure sooner than the corresponding non-PEMF-treated mice. For the PEMF-treated, the average full closure time for the wild type was 11 ± 2 days, and for the diabetes mice, it was 16 ± 4 days. Contrastingly, for untreated mice, it was 15 ± 3 and 24 ± 5 days, respectively. Based on additional measurements including wound bed blood perfusion, vascularity and fibroblast growth factor (FGF-2), the authors suggested a linkage between pulsed electromagnetic field therapy (PEMFT) and upregulation of FGF-2, promoting angiogenesis and increased cellular motility. In a separate study, wounds were created by making a 35 mm dermal incision on the right side of the paravertebral region of STZ-induced diabetic and non-diabetic rats [17]. PEMF was created by a function generator connected to copper coils that resulted in a 4 ms pulse train at 20 Hz producing an 8 mT PEMF signal. Rats were divided into four groups: non-treated control, PEMF-treated control, non-treated diabetic, and PEMF-treated diabetic. Treated rats were exposed to PEMF for one hour daily for 16 days, while non-treated groups were not exposed to PEMF. Percent wound closure was assessed on days 0, 4, 8, 12, and 16 after wound induction. Results showed a statistically significant improvement in healing rate in PEMF-treated rats and a reduced time to wound closure in non-diabetic rats (p<0.01) and in diabetic rats (p<0.001).

Other findings have less support for the use of PEMF to accelerate wound healing based on the STZ-induced diabetic rat model [18]. Seven days after STZ injection, 2 cm x 2 cm full-thickness square wounds were made on the rat’s back and then randomly divided into a PEMF-treatment group (n=28) or a control group (n=28). PEMF-treatment was done using a commercial device (Model XKC-600W Magnetopulse International, Griffin, Australia). This device produces sinusoidal pulse trains at 25 Hz with a pulse width of 40 ms and a peak intensity of 5 mT. Treatment was one hour per day with measurements made on days 0 (wound induction), 7, 10, 14, and 21 post-wounding. Examination of their data indicates some minor improvements in early healing rate with PEMF-treatment but essentially no difference in endpoint (21-day) wound area closures, being on average about 99% closed for each group. Subsequently, the same rat model was used to evaluate the effect of PEMFT on collagen during wound healing in 20 PEMF-treated vs. 20 non-PEMF-treated control rats using the same PEMF treatment parameters [19]. Rats received treatment for one hour daily with measurements made on days 7, 10, and 14 post-wounding. At the seventh-day evaluation, the PEMFT group had a higher amount of type I collagen deposition (p=0.013) that continued to increase through day 14, but due to wide standard errors in values, differences between groups beyond seven days did not prove to be statistically different. There were also no significant between-group differences for collagen fibril alignment and collagen fiber orientation. A recent study provided strong evidence supporting the role of SMF as a modality that accelerates wound healing in a diabetic mouse model that had experimentally induced 5 mm punch biopsy back wounds [20]. They pre-treated genetically obese leptin receptor-deficient db/db diabetic mice with seven weeks of continuous SMF exposure of about 15 mT at the wound site and continued treatment for three weeks post-wounding. Wound closure percentages were determined at 4-, 9- and 22-days post-wounding. Similar procedures were done to db/db mice exposed to sham magnets and also to normal non-diabetic mice as controls. At the 22-day assessment, wound closure for the sham-treated group was about 65%, whereas for the SMF-treated groups it was about 90% (p<0.001).

Another study used PEMF to treat a small number of patients with diabetic foot ulcers [21]. In this study, 13 patients with diabetes-related foot ulcers were treated with a commercial PEMF device (Model XKC-600W Magnetopulse International, Griffin, Australia). This device produces sinusoidal pulse trains at 12 Hz with a pulse width of 40 ms and a peak intensity of 1.2 mT. Patients received 14 PEMF treatments (n=7) or 14 sham treatments (n=6), each lasting one hour over an interval of three weeks. The results of this small pilot study indicated that PEMFT did not significantly decrease wound area but may have decreased wound depth in the PEMF treated group. Diabetic foot ulcers have also been treated with a novel PEMF device dubbed therapeutic magnetic resonance (TMR) [22]. This device was reported to generate a complex pulse train with varying frequencies and polarities and an average generated field intensity of 40-60 mT, was used to treat 20 patients with sham treatments of 20 others. The reported outcome of active treatment was an increase in granulation tissue components that included collagen and integrins, along with a reduction in pro-inflammatory interleukins and increased growth factor expression.

Diabetic Neuropathy

Detailed aspects of diabetic neuropathy have recently been extensively reviewed [23] and its impacts on patients' daily lives have been described [24]. Various methods for its pain-related mitigation have also been reviewed [25], and PEMF as a possible treatment has been suggested [26]. In this section, the potential role of EMFT on pain-related aspects is considered. In experimental work to assess treatment-related changes, two commonly used assessment parameters are allodynia and hyperalgesia because of their presence in persons with diabetic neuropathy [27]. In this context, allodynia describes pain caused by a stimulus that would not ordinarily cause pain, and hyperalgesia describes heightened pain to a stimulus that would ordinarily cause lesser pain. 

Using a STZ-rat model, the effects of PEMFT on allodynia and hyperalgesia were evaluated using PEMF spiked pulse trains of different frequencies with peak field intensities of 1.5 mT [28]. The treatment pattern used repeating four-minute bursts of impulses alternating between either 1-Hz and 5-Hz or between 30-Hz and 40-Hz for an overall treatment interval of 60 minutes. Animals were treated for five weeks with weekly evaluations. Allodynia and hyperalgesia were evaluated with a thermal plantar test that measures paw withdrawal time and a device that uses a touch-stimulator to measure the force at which paw withdrawal occurs [29,30]. Allodynia was assessed based on the paw-withdrawal threshold to a light touch of the hind paw. The severity of hyperalgesia was assessed by paw withdrawal latency to thermal stimulation. Induction of STZ-diabetes was associated with an increase in both allodynia and hyperalgesia, but the 1-Hz and 5-Hz treatments significantly blunted both of these changes (p<0.05). There were minimal effects of the 30-Hz and 40-Hz treatments. Subsequent work using this rat model used a similar treatment pulse train pattern now consisting of one repeated sequence of 1, 3, 5, and 7 Hz also delivered over four minutes with rats treated daily for one hour/day for four weeks [8]. After two weeks of PEMFT, allodynia and hyperalgesia measures statistically improved by 11% and 15%, and by four weeks, both were restored to near non-diabetic levels. A parallel sham-treated group showed no significant improvement. A slightly different PEMF pattern, consisting of pulse trains of 1.5 mT peak delivered at 10-Hz or 30-Hz, was used to assess neuropathy pain mitigation and biomarkers of PEMFT changes [31]. Both patterns improved allodynia and hyperalgesia measures, with the 10-Hz pattern being more effective and sham treatment producing no improvement. In addition to pain parameters, certain genes were determined using polymerase chain reaction (PCR) before and after STZ induction and during PEMF treatment. It was reported that the gene (SCN11A) that codes for the voltage-gated sodium channel NaV1.9 were reduced after neuropathy induction but that PEMF 10-Hz treatment brought it back to near-normal levels. The authors concluded that PEMF 10-Hz therapy may reduce pain by modulating voltage-gated sodium channels at the level of transcription and that 10 Hz can more effectively manage pain than 30-Hz PEMF treatment.

Several studies have used various forms of PEMFT to try to mitigate diabetic neuropathic pain in patients. In one, use was made of a bed-shaped device (Viofor JPS device, Med & Life, Komorow, Poland) [32]. Patients could lie on the device and be exposed to a complex PEMF signal consisting of frequencies between 180 and 195 Hz with a reported field intensity of up to 100 mT. PEMF treatment was given to 32 patients with a starting average visual analog pain score (VAS) of 73 mm, and sham treatments were given to 29 patients who had an average starting VAS of 69 mm. After five weeks of PEMF or sham treatment, both groups reported significant decreases in VAS to 22 mm and 44 mm, respectively, with no statistical difference in reductions between groups. Another study used VAS scores to evaluate the impacts of PEMF treatment in 24 patients with refractory foot neuropathic pain. The treatment consisted of a patented priority unspecified pulse sequence of frequencies near 30 Hz and a peak field intensity of 2 mT delivered to the soles of the feet during nine one-hour treatments over a two-week interval [33]. The outcome was reported as a significant decrease in average pain score from a pretreatment value of 6.26 cm to 3.96 cm, assessed four weeks after the end of treatment (p<0.01). It should be noted that this study did not focus explicitly on patients who were diabetic, and there was no sham control used. However, the author suggested that the pain reduction might be related to the PEMFT causing either repolarization or hyperpolarization of sodium channels associated with unmyelinated c-fibers or small A-delta nociceptors located within the epidermis and dermis of the treated foot.

Blood Glucose

The effects of 200, 400, and 600 mT SMF on blood glucose in type 1 and type 2 diabetic mice were evaluated via continuous 60-day treatment [34]. Type 1 diabetes was induced using alloxan and a high-fat diet, whereas type 2 diabetes was STZ-induced. Treatment was delivered via multiple neodymium magnets or sham magnets placed in the bottom of the cage in which mice were housed. Blood glucose changes were measured on days 30 and 60 of treatment in response to an intragastric dose of starch. Blood glucose levels were measured at standardized times after starch administration. On day 30, mice with type 1 diabetes who were treated with 400 mT showed a statistically significant reduction (p<0.05) in blood glucose compared to sham-treated mice. On day 60, all treatment groups showed statistically significant reductions (p<0.05). For mice with type 2 diabetes, glucose was significantly reduced (p<0.01) only for mice treated with 600 mT. A similar reduction trend for the resting blood glucose of STZ-induced diabetic mice was reported for mice housed in cages and exposed to nonhomogeneous fields for 30 minutes/day for six weeks [35]. The SMF-treated diabetic mice were exposed to about 477 mT at their feet and about 2.8 mT at the top of their heads. A corresponding group was sham-treated. At six weeks, both groups had elevated blood glucose levels compared to non-STZ treated mice, but compared to sham-treated diabetic mice, the magnet treated had a significantly reduced blood glucose level (p<0.001). 

The potential effect of the direction of the magnetic field on blood glucose was also investigated when treating STZ-induced diabetic mice [36]. Four groups were considered: a sham group, an average upward field (≈100 mT), an average downward field (≈100 mT), and an alternating upward and downward pattern producing between 40 and 50 mT. Six mice per group were treated for two hours/day for 12 weeks. Downward SMF treatment reduced fasting blood glucose levels vs. sham-treated (p<0.05) and also improved intra-peritoneal glucose tolerance test results vs. sham-treated (p<0.05). In contrast, upward SMF treatment decreased glucose clearance vs. control (p<0.01), thereby indicating a negative treatment effect on hyperglycemia.

A novel approach was undertaken to study the effects on fasting glucose and glucose tolerance when mice were exposed to the SMF aligned mainly along the long axis of mice combined with an electric field aligned perpendicular to their long axis when housed in non-magnetic cages [37]. A major finding reported was improvements in both glycemia and glucose tolerance only when both magnetic and electric field exposure were present. No effects or even negative effects were present when either alone was used. In a series of related experiments, these researchers suggested that the various beneficial effects of the combined fields on insulin resistance were likely at least in part attributable to reaction product modifications in line with the concept of field-induced radical pair mechanisms [38-41]. Subsequently, an alternate explanation was put forward in which it was hypothesized that the combined magnetic and electric fields affected the vestibular system via modifications of inner ear endolymph currents [42]. It was suggested that this triggered a stress response with an associated increase in catecholamines and adenosine monophosphate (AMP)-activated protein kinase, both of which can decrease insulin resistance and decrease hyperglycemia. This view was considered unlikely since the magnitude of the fields used in the original study were not large enough (3 mT and 7 kV/m) to explain the insulin-sensitizing effects originally reported [43]. The effects of PEMFT on blood glucose have also been investigated in the STZ diabetic rat model [44]. The primary focus was on diabetic neuropathy symptoms of hyperalgesia and allodynia, but they also reported ameliorating effects of PEMF on blood glucose. After four weeks of PEMF exposure, blood glucose levels decreased by a mean of 15% (p<0.05), but sham-treated rats experienced no significant change.

Blood Flow

There is substantial evidence of the involvement of microcirculatory deficits in diabetes [45-50]. It is thus useful to consider the reported effects of static and time-varying magnetic fields on blood flow that may indirectly provide insight into potential linkages to diabetic therapy targeting microvascular deficits. Measurements of human skin blood flow (SBF) when hands or fingers were exposed to a perpendicular SMF have yielded varying results, with SBF reported to decrease [51], not change [52-54], or show increased vasomotion-related changes [55]. Other work using SMF on experimental animals has also reported varying results, with SMF exposure causing blood flow to decrease [56,57], not change [58], increase vascular diameter [11], and increase or decrease vascular diameter depending on their basal state [59], and cause alterations in microvascular vasomotion patterns [60,61]. Other aspects of SMF-related impacts on microcirculation in relation to diabetes and wound healing have been presented [62-64]. The effects of time-varying or pulsed electromagnetic fields on blood flow have also been documented as having varying effects on skin blood flow. Exposure of hand and finger skin to field intensities between 32 and 48 mT at 3.8 kHz caused a transient decrease in blood flow lasting about 10 seconds but no sustained change in blood flow [65]. Contrastingly, there have been reports suggesting a positive angiogenic role of PEMFT and an increase in blood flow in an experimental ischemic skin model [66,67]. Other reports suggest no blood flow effects attributable to specific forms of PEMFT [68]. It is likely that further research is needed to pin down the role of EMFT as an effective blood flow modulator and to define the conditions to which it is applicable.

However, there are some aspects of its role in the treatment of diabetes conditions that are of relevance. In a small study (n=7 treated and 6 sham), diabetic plantar ulcers were treated with 12 Hz pulsed fields, stated to achieve an intensity of 1.2 mT. Compared to the sham-treated outcomes, these workers reported improved wound healing along with an increase in capillary red cell velocity measured on the great toe dorsum associated with the PEMF treatment [21]. However, the small number of patients in this study and the absence of experimental details as to the placement of the treatment device, suggest these findings should be interpreted cautiously. A more clearly defined study used a similar 12-Hz signal with an intensity of 0.5 mT to evaluate PEMF treatment effects on small superficial veins of the foot and great toe skin blood flow in 22 persons with diabetes and 21 persons free of diabetes as controls [69]. They report increases in blood velocity in small veins in both diabetic and healthy persons, but no such increase with sham treatment. Based on their data, an average velocity increase of 26% and 27% was calculated for the diabetes group and controls, respectively. Further research will be needed to more clearly characterize the potential of EMFT in blood flow modification.

Inflammation and Oxidative Stress

Inflammation is often a component or complication of diabetes, so it is of value to examine studies that have investigated EMFT's effects on inflammatory markers. One such study, already discussed from its wound healing outcomes, also reported the role of SMF treatment in resolving inflammation associated with wound healing [6]. Results of immunofluorescent staining indicated that SMF treatment accelerated wound healing by shifting macrophage polarization toward the M2 phenotype in comparison to M1. This was thought to occur by upregulating anti-inflammatory gene expression in STAT6 while suppressing pro-inflammatory STAT1 in macrophages.

Results from studies using PEMF have also yielded relevant information regarding the impacts of EMFT on inflammatory processes. Using a 50-Hz, 7 mT peak intensity sinusoidal signal as a seven-day treatment for Wistar rats revealed that plasma levels of various pro-inflammatory cytokines depended on whether treatment was delivered one-hour/day or continuously for 24-hours [70]. In comparison to controls, continuous exposure but not one-hour repetitive treatment significantly increased interleukins IL-1B, IL-2, and IL-6 (p<0.001). PEMF-related changes in the inflammatory-mediators defensin and C-reactive protein (CRP) were investigated in 32 patients with diabetic painful neuropathy using the same commercial bed-shaped device previously described (Viofor JPS, Komorow Poland) [71]. As noted, this device generates a 100 mT intensity field using a complex pulse train varying between 180 and 195 Hz. Treatment was given for 20 minutes/day for 15 days over three weeks with no significant effects on either CRP or defensin in the diabetic patients. Others have suggested that SMF may have a role in mitigating oxidative stress [72].

Summary discussion

The summated result of the present investigation indicates both successful and unsuccessful applications of EMFT as applied to the diabetic condition. There is some evidence for potentially useful outcomes for diabetic wounds, neuropathic pain, inflammation, blood glucose levels, and possibly blood flow. In animal models, lower frequency EMFTs appeared to have a greater effect in treating symptoms of neuropathy. For future human trials, it will be important to see if this finding is similar. With the increasing prevalence of diabetic complications, EMFT may potentially be considered as an innovative and cost-effective alternative to the standard management of diabetic complications [26]. Pharmaceutical approaches are commonly used to suppress immune system responses and inflammation to treat diabetic complications, but may be costly and may hinder wound healing. Contrastingly, it has been reported that SMF treatment may positively influence wound healing and tissue regeneration by balancing the signaling of the pro-inflammatory gene STAT1 with the anti-inflammatory gene STAT6, thereby reducing inflammation [6].

The benefit of SMF is also potentially useful in the treatment of other diabetic complications as reported for improvements in blood glucose levels, neuropathy scores, and tissue perfusion via vascular vasodilation. SMF treatment has also been reported to improve healing rates, reduce healing time, and increase tensile strength in diabetic wounds [10,15]. SMF treatment may also reduce burning, numbness, tingling, and foot pain in cases of diabetic neuropathy. When treated with combined SMF and electric fields, an important effect on insulin sensitivity was reported that was not present for either treatment modality alone [37]. While the mechanism behind this improvement is unclear, it would appear that this avenue of research is worthy of pursuit. PEMF treatment in some studies had directionally similar effects as SMF in diabetic wounds and was reported to decrease wound-healing time and improve the quality of granulation tissue [12,22]. PEMF treatment also increased type 1 collagen deposition, which promoted wound healing [19] and upregulated FGF-2, which may be an important factor in facilitating wound healing. In animal models of diabetic neuropathy, PEMF had anti-allodynic effects [8]. Additionally, the beneficial effects of SMF and PEMF were also reported in human trials [9,33]. However, further studies are needed to confirm. There have also been studies that suggest EMFT therapy is not as effective. In a double-blind study, subjects with polyneuropathy were given PEMF over the course of three weeks [71]. The results showed that there was no reduction in CRP or defensin levels two weeks following treatment. Another study looked at the effects of EMFT on VAS scores and found no difference between sham and PEMF-treatment subjects [32]. These negative studies further support the need for more research to investigate EMFT therapy as a viable treatment option for diabetes conditions.

## Conclusions

Reports from both animal and human studies provide support for the adjunctive use of EMFT, including both SMF and PEMF, to address complications of diabetes, including wounds, chronic pain, and neuropathy. These non-invasive modalities show promise, with no known reports of untoward effects. Currently, there are insufficient high-quality systematic studies on humans to provide high levels of confidence in such treatments, and as such, it would appear prudent that utilization of EMFT for diabetic-related complications should be used in conjunction with the standard of care. If such care is not easily available, however, EMFT may serve as an interim single therapy. Further research with specific targets of this modality could provide additional understanding and confidence in this potential treatment modality. Finally, it should be emphasized that while many studies showcase positive attributes of EMFT, the biological pathways behind these reported outcomes have yet to be discovered. Going forward, the mechanisms behind the use of EMFT to treat diabetic and potentially other conditions appear to be a worthwhile goal to help mitigate the worsening diabetic complications seen as a result of the COVID-19 pandemic.

A key limitation of EMFT is that the mechanism of action is not understood. Multiple studies have shown positive results. However, more research should be done to discover how EMFT is able to improve diabetic complications and what its limitations are. Most studies conducted have also occurred in laboratory settings or using animal models, illustrating the need for further clinical studies before implementing EMFT in humans.
